# The Impact of Internet and Videogaming Addiction on Adolescent Vision: A Review of the Literature

**DOI:** 10.3389/fpubh.2020.00063

**Published:** 2020-03-05

**Authors:** Ioanna Mylona, Emmanouil S. Deres, Georgianna-Despoina S. Dere, Ioannis Tsinopoulos, Mikes Glynatsis

**Affiliations:** ^1^2nd Department of Ophthalmology, School of Medicine, Aristotle University of Thessaloniki, Thessaloniki, Greece; ^2^Ophthalmology Department, “Hippokration” General Hospital of Thessaloniki, Thessaloniki, Greece; ^3^“Asclepius” Private Mental Health Hospital of Veroia, Veria, Greece

**Keywords:** internet addiction, videogame, gaming disorder, vision, digital eye strain

## Abstract

During the past decade, vision problems that were attributed to the use of electronic screens have gradually shifted from being a workplace health issue to a wider public health issue. “Computer vision syndrome” originally related to the few professionals exposed to long hours of work in front of a computer screen. The widespread use of digital screens in devices used throughout the day have led to the emergence of “digital eye strain” as a new clinical syndrome that affects every individual who spends a large period of time fixated on multiple screens, for work or leisure. A new subcategory, “video game vision” has been proposed to specifically address vision issues related to large periods of continuous use of screen enabled devices in order to play video games. With gaming disorder being included in the next version of the WHO classification of diseases (ICD-11), it is becoming increasingly important to have a clear idea of the impact of this disorder in general health and functioning. At the same time, a number of research studies have reported positive impact of videogame playing on the players vision. This article reviews the latest research studies on the impact of digital screen enabled devices on adolescent vision in light of the increasing reports of internet addiction and gaming disorder while referencing positive findings of videogaming on vision in order to provide a balanced approach and assist with classification, diagnosis and treatment, while providing directions for future research.

## Introduction

During the past decade, vision problems that were attributed to the use of digital screens have gradually shifted from being a workplace health issue to a wider public health issue (1). “Digital eye strain–DES” (2) is a newer term, coined to include all aspects of eyesight issues related to long hours of work in front of a digital screen.

A number of professional bodies are providing guidance for practitioners with regards to vision issues related to digital screen viewing (3). However, these available guidelines tend to overlook important reasons for vision-related symptoms from prolonged exposure to screen viewing: Internet addiction and gaming disorder.

Research on online and offline addictive behaviors has led to the subcategory of gaming disorder being included in the forthcoming version of the WHO classification of diseases, ICD-11 (4). A related classification, “videogame vision syndrome” has been proposed to specifically address vision issues related to large periods of continuous use of screen-enabled devices in order to play videogames (5).

However, it is worth noting that there are well-documented findings that videogames improve vision as a whole (6) and these are frequently referenced as a counter-argument on the negative impact of videogaming (7). Since it is becoming increasingly important to have a clear picture of the full impact of internet addiction in general, and gaming disorder in particular, on general health and functioning (8), this review focuses specifically on related problems with vision.

## Materials and Methods

We have opted for a narrative review in order to determine whether the available body of knowledge on DES also addresses the negative effect of gaming disorder and internet addiction on vision, with an emphasis on adolescent participants. The scope was expanded to include reports on positive effects on vision from videogaming, a brief overview of specific eyesight symptoms, along with diagnosis and treatment suggestions. We reviewed available studies from online databases including PubMed, PsychINFO, ProQuest, and EMBASE on suitable material. A total of 246 articles were retrieved and reviewed for relevancy in the first wave of search that only included negative aspects on vision, yet only 12 were directly related to the theme. References of those articles deemed suitable were also explored for relevant studies.

## Results

### Negative Effects of Videogaming, Problematic Internet Use, and Gaming Addiction on Vision

Nearly all the retrieved studies did not attempt to differentiate their subjects according to the activities while viewing digital screens. The outcome measures were mostly self-reported symptoms and not objectively measured findings. The issues that were reported were related either to with prolonged near-term adaptation (i.e., blurred vision at close range, difficulty in focusing, and copious headache after screen use) and those related to dry eye syndrome (irritation/burning sensation, ocular fatigue, discomfort, photosensitivity), while symptoms due to poor posture and prolonged physical immobilization in front of the screen (such as neck pain, tension headache, and other atypical musculoskeletal pain) are also very common. Pre-existing vision problems (hyperopia / myopia, astigmatism, and adaptive disorders) can contribute to the appearance of the syndrome if they are not adequately addressed or have not yet been diagnosed (9, 10).

A review of 38 studies of problematic internet use among students from the Southeast Asia, a region with high reported prevalence of internet and gaming addiction, reported that 19% of the subjects experienced eye strain (11). This percentage compares to the findings of a recent meta-analysis on the incidence of DES in pediatric populations that reported a pooled prevalence of asthenopia as being 19.7% (12.4–26.4%) (9).

Studies in South Korea demonstrated that time spent watching content in any digital screen (12) and especially in a smartphone (12, 13) are risk factors for DES in adolescents. Using smartphones for more than 2 h daily is associated with a higher incidence of multiple DES symptoms (13). Studies on the impact of exposure time to digital screens on children demonstrate that the chances of symptoms increase after 2–4 h of exposure (13, 14). A qualitative research article presented results from focus groups and interviews conducted with 368 children between 9 and 16 years old in nine European countries on what they perceived as being potentially negative or problematic while using the internet and technology (15). Children reported a variety of eye problems, such as their eyes hurting, eyestrain, and needing to wear glasses due to prolonged internet usage. A recent controlled study enrolled fifty healthy college students to play an online videogame on their computers continuously for 4 h in the night (16). Results demonstrated convergence and accommodation disturbances and increased physical and ocular discomfort while near phoria showed an exophoric shift and the accommodative and vergence facilities along with blink rate were significantly decreased. Distance phoria remained unchanged and all visual functions recovered to the baseline levels by the following morning.

Ergonomic practices carry an even more significant impact on the occurrence of eye fatigue in adolescents and young adults, compared to 30 years ago. With the newer handheld devices, the confines of a desk no longer exist, along with the appropriate ergonomic measures that can be applied. Low ambient illumination creates fatigue for the pupil's reflex, while a small proportion of the font and screen size results in faster adaptation fatigue. In South Korea, students aged 7–12 years, who used a smartphone for more than 4 h a day over a period of at least 4 months, presented with acute acquired comitant esotropia (17). A study in India (18) included 576 adolescent students and reported a significant correlation in the practice of lying down while using a screen-enabled device to symptoms of digital eye strain (27%). By the end of the day 18% of the students experienced eye strain, attributed to digital screen use. Eye strain (67%), dry eyes (26.2%), double vision (28.9%), and blurred vision (51.6%) were common symptoms in a survey of 409 Medical college students in Jamaica (19). Eye burning and eye strain were significantly related to level of viewing. Moderate eye burning (55.1%) and double vision (56%) occurred in those who used handheld devices.

A more objective measurement is visual acuity. There haven't been consistent reports on a direct negative impact on visual acuity and the review of DES referenced earlier concluded that the majority of children with asthenopia did not present visual acuity or refraction abnormalities (9). A large scale epidemiological survey of data from 1979 to 2012 in Japan on the proportion of students with poor visual acuity concluded that there was only a weak negative correlation of videogame duration and visual acuity (20).

In the referenced literature up to that point, there was no attempt to discern the clinical presentation of eyesight issues related to gaming addiction from any other use of screen enabled devices. However, the need for experimental research on the wider question as to whether gaming addiction can be meaningfully separated from other types of digital screen use with regards to the negative effects on vision does exist, as demonstrated by a recent study in Italy that examined two groups of children aged 3–10 years as to the average amount of time spent playing videogames daily (5); children who played videogames for <30 min per day and not every day (control group) and children who played videogames for 30 min or more every day (videogame group). Those groups were controlled for the use of other types of digital screens. Symptoms of eye strain, including headache, eyelid tic, transient diplopia, and dizziness, absence of fine stereopsis, and refractive errors were statistically more frequent in the videogame group and persisted in the comparison regardless of general use of digital screens.

So far, the only experimental study to explicitly compare adolescent videogame players to other adolescent non-players who used screens for different purposes was carried out almost 30 years ago in Japan by Misawa et al. results indicating that the eye movements during videogames were more rapid and frequent than those during conventional work on screen while the viewing distance between the eyes and the TV screen was shorter for videogames (on the TV) than for watching TV programs. No significant difference was found in the decrease of critical flicker fusion and the extension of near point distance compared to a word processing task and a videogame (21).

### Positive Effects of Videogaming on Vision

Players have presented better results than non-players in a variety of tasks that involve vision but are ultimately controlled by cortical structures in the brain, especially those involved in prediction. These include enhanced contrast sensitivity (22), shorter saccadic reaction times with better error rates (23), higher spatial resolution of vision (24), and a variety of specific improvements on memory function and focused attention. Playing action videogames can alter fundamental characteristics of the visual system, seen as a whole, that is, including the cortical structures that are responsible for image processing, pre-emptive movements of the ophthalmic muscles. This is reflected in findings of a robust positive association between cortical thickness and videogaming duration was observed in left frontal eye fields which are a key region involved in visuo-motor integration important for programming and execution of eye movements and allocation of visuo-spatial attention, processes engaged extensively in videogames (25).

Although these positive findings have been employed clinically to guide videogame use in order to enhance innate vision deficiencies or even assist with surgeon training, they should not be a reason to blindly promote videogame use; Cognitive gains do not correlate with loss of functionality in the vision sensory organ, which is the eye. Cognitive gains have been demonstrated in all test designs with relatively few hours of playing videogames, and as with all aspects of brain plasticity, gains are to be expected with frequent execution of a well-designed task that lasts for relatively little time (26). Hence, playing random videogames to the degree that it may cause temporary harm to the receptor organ does not correlate positively with gains in the vision process as a whole. The increased propensity of eSports during the past decade, i.e., videogaming as a form of sport competition could be a useful source of research data; playing competitively does not equate with playing excessively, yet those competitive players need to regularly exercise their skills, even spending 6 h daily of deliberate practice and various forms of non-deliberate practice that revolves around viewing others play through a digital screen (27). Unfortunately, published research on this niche population so far is poor. An anonymous survey of 65 collegiate eSport players from nine universities across the USA and Canada who practiced between 3 and 10 h per day showed that the most frequently reported complaint was eye fatigue (56%) (28).

## Discussion

### Reporting Vision Complaints Associated With Gaming Disorder and Internet Addiction

There was a paucity of relevant studies on negative effects of gaming disorder on vision; vision symptoms are typically included as non-specific symptoms in a list of self-reported somatic symptoms and not objectively measured and quantified.

### Directions for Future Research

Future research needs to specifically compare sub-populations of gaming addicts to non-addicted gamers who spend a comparative amount of time in front of a digital screen. eSports players are an interesting subpopulation in this respect, since they strive for competitive gaming and not addictive involvement, yet still focus intensely on their gaming.

The objectively measured parameters that need to be researched relate to all possible mechanisms of induced eyesight issues. Ocular motility problems with prolonged exposure to screens are a consequence of a proximal reflex disorder, with the ability to adjust the eye found considerably burdened by even only 60 min of uninterrupted screen use (29). Pupillary response is an important parameter of close vision with problems reported in up to 33% of people after intense close-eye work as the pupil may maintain a contraction state after work is completed (30). Prolonged goal-oriented work on a computer screen resulted in a reduction in maximum adaptability and speed as well as a reduced response to light exposure by delaying the pupil's direct reflex, reduced range, and maximum diameter, all signs attributed to uncoordinated stimulation of the sympathetic and parasympathetic system after fatigue of the reflex (31). Dry eye problems associated with DES are associated with a reduced blinking frequency. Blinking helps maintain a normal eye surface by intensifying a cycle of tear secretion, dispersion, evaporation and drainage (32). Increased blinking occurs due to low readability conditions that increase the duration of focus and require increased time to obtain visual information. A study on visual fatigue induced by a tablet computer found a significant decrease in tear film break-up time (TBUT) but no difference in ocular wave-front aberration (33). This is the only study on issues with recently-developed screens and more research is needed to assert whether newer screen technology may ameliorate the negative impact on vision.

Sleep deprivation associated with screen viewing is another symptom that has not been researched. Problems due to exposure to blue light are a consequence of the newer forms of light emitting large amounts of blue light (440–500 nm), leading to photochemical damage (34). Research has also confirmed that screens of digital devices may interfere with children's' sleep due to blue light emission (35), which suppresses melatonin production (36), negatively affecting circadian rhythms and cognitive performance (37). This leads to a vicious circle of poor sleep hygiene due to excessive use of screen-enabled devices that is augmented by the circadian rhythm disruption (38). Most videogamers indulge in large periods of play precisely during night time, since night playing can offer longer uninterrupted duration of play and is conducive to game genres which need dedicated time to organize multi-playing (39). Adolescents will have completed their study workload for the following day or simply afford to spend all this night time because the parental monitoring is easier to avoid. Using glasses that block low wavelength radiation at night. can improve sleep duration and quality, while reducing subjective alertness (40).

### Assessment of Vision Complaints in Suspected Cases of Internet Addiction and Gaming Disorder

An important handicap in the clinical assessment is the fact that children may ignore vision related complaints if they are enjoying what they do with their screen-enabled device (41). Children also tend to underreport symptoms that do not impede viewing completely, like dry eye, although they will report symptoms that impeded viewing, like haziness (42). A reliable estimation of time spent using screens is thus of paramount importance and it should include specific measures of all types of screen-enabled devices. A validated questionnaire on gaming addiction could be helpful in this respect (43).

### Addressing Cases of Vision Issues in Internet Addiction and Gaming Disorder

Reducing screen time is helpful especially in cases where there is limited parental guidance or control. Monitoring the usage time can reveal a pattern of chronic sleep difficulty due to preoccupation with screen enabled devices that is exacerbated by nighttime use and the associated circadian dysfunction. A reduction in smartphone use during 4 weeks in children with DES aged 7–12 years lead to improvement in all parameters that examine ocular surface, like the tear break-up time, number of punctate epithelial erosions and the ocular surface disease index, with all children no longer affected with DES anymore by the end of the 4 weeks (13).

Lens with blue light filters may assist with sleep disturbances and protect from phototoxicity (44). However, any such provision to the adolescent should not carry the implicit message that it is now acceptable to play during late hours, since circadian disturbances will persist when basic sleep hygiene is neglected.

Symptomatic treatment includes eye drops with 2% povidone which decreased symptoms of ocular tiredness, dryness, and difficulty of focus while maintaining an unchanged corneal surface and improving dynamic visual acuity in contact lens wearers with computer vision syndrome, although all symptoms were not fully resolved (45). A study also demonstrated a reduction in signs and symptoms of dry eye after dietary intervention with omega-3 fatty acids, with 70% of cases being symptom-free after 3 months (46).

## Conclusions

Eye symptoms related to excessive use of screen enabled devices due to internet addiction or gaming disorder have been underreported in the relevant literature. A small number of studies have demonstrated that these symptoms may be more pronounced than symptoms of DES related to other reasons for using digital screens. More research is required in order to ascertain the etiology of those differences between different types of use, so as to provide parents and users with concrete, evidence-based guidelines. In contrast, positive aspects of playing videogames on vision have been extensively researched and widely reported, yet they refer to cortical structures that are indirectly trained to perform better on specific task-oriented functions, as is the case with any aspect of brain plasticity. A more balanced approach is required, considering the wider public health impact of gaming disorder. Assessing not only the total time spent using any kind of digital screens, but also the reason for doing so, can be important for planning treatment of both eyesight and behavioral problems.

## Author Contributions

IM, ED, and G-DD contributed to the data acquisition and analysis. IM and MG contributed to the concept and design of the work. IM drafted. ED, G-DD, and IT revised the manuscript. MG supervised the work. All authors agree to be accountable for the work.

### Conflict of Interest

The authors declare that the research was conducted in the absence of any commercial or financial relationships that could be construed as a potential conflict of interest.
